# Treatment with Mirvetuximab-Soravtansin—first clinical experience in advanced ovarian cancer in a comprehensive cancer center

**DOI:** 10.3389/fonc.2026.1732304

**Published:** 2026-02-19

**Authors:** Isabella Schwörer, Sarah Huwer, Beate Rautenberg, Jakob Neubauer, Ingolf Juhasz-Böss, Lisa Jung

**Affiliations:** 1 Department of Obstetrics and Gynecology, Medical Center – University of Freiburg, Faculty of Medicine, Freiburg, Germany; 2 Department of Diagnostic and Interventional Radiology, Medical Center – University of Freiburg, Faculty of Medicine, Freiburg, Germany

**Keywords:** advanced ovarian cancer, folate receptor α (FRα), Mirvetuximab-Soravtansin, ocular toxicity, platinum-resistant

## Abstract

**Introduction:**

Mirvetuximab-Soravtansin (MIRV) is an antibody–drug conjugate (ADC) for the therapy of advanced platinum-resistant folate receptor α (FRα)-expressing ovarian cancer. The goal of the study was to treat patients with MIRV off-label in our Comprehensive Cancer Center regarding the outcome and adverse events.

**Methods:**

This retrospective single-center cohort study included six patients with advanced high-grade serous ovarian cancer (HGSOC) or high-grade serous primary peritoneal carcinoma (PPC) with initial FIGO stage IIC–IV between July 2023 and August 2024. Inclusion criteria were metastatic advanced HGSOC or PPC, expression of FRα ≥75% (IHC PS2+), and progression during systemic therapy with ≥3 therapies before. All patients underwent ophthalmological examination before therapy with MIRV.

**Results:**

After 2–6 cycles of MIRV (median, 3.3), objective descriptive response with computed tomography (CT) scan of the thorax–abdomen and CA-125 response were monitored. CA−125 decreased in all evaluable patients (*n*, 5) from baseline. In the CT scan, two patients showed radiographically stable disease, and one of our patients represented progressive disease. Two patients revealed a partial response, and one of them had partial remission at the initial FIGO stage IV. Adverse events were monitored; one patient developed transient ocular toxicity.

**Discussion:**

Treatment with MIRV has been shown to be promising in advanced ovarian cancer. In a small heterogeneous group of heavily pretreated patients, general recommendations for therapy with MIRV are limited. Close collaboration with ophthalmology and patient education is essential to mitigate ocular events.

## 1 Introduction

Mirvetuximab-Soravtansin (MIRV) is an antibody–drug conjugate (ADC) for the therapy of platinum-resistant ovarian cancer (1). MIRV targets folate receptor α (FRα), which is overexpressed on advanced ovarian carcinomas (2, 3).

Mirvetuximab acts as an antibody of FRα, and Soravtansin functions as a tubulin-targeting agent (maytansinoid DM4) (4).

Ovarian cancer is one of the deadliest gynecologic cancers, second only to breast cancer in mortality. A total of 7,180 new cases of ovarian cancer occurred in Germany in 2020 (5). Worldwide, more than 300,000 newly diagnosed cases of ovarian cancer occur every year (6). Most of these are diagnosed in stage III/IV (73%). The 5-year survival rate decreases from 90% in stage I to 21% in stage IV (5).

Primary treatment of ovarian cancer should be surgery with R0-resection (7, 8). In advanced ovarian cancer (FIGO stage II–IV), additional adjuvant chemotherapy with six cycles of carboplatin AUC 5 and paclitaxel 175 mg/m² q3w is recommended (8). Stage III and stage IV should receive maintenance therapy with PARB-inhibitor ± bevacizumab depending on HRD (homologous recombination deficiency) and BRCA status.

Unfortunately, most ovarian cancer relapses in the first years after primary treatment. Platinum-sensitive ovarian cancer shows primary response to platinum-based therapy and relapse after more than 6 months. Platinum-resistant ovarian cancer returns during the first 6 months after initial chemotherapy with carboplatin. Platinum-resistant ovarian cancer treatment consists of mono-chemotherapy without platinum, e.g., gemcitabine, paclitaxel weekly, pegylated liposomal doxorubicin, or topotecan. Patients with platinum-sensitive ovarian cancer could receive either surgery to try for R0-resection or platinum-based polychemotherapy (8).

After the first relapse, most patients develop recurrent disease with poor response to chemotherapy (9, 10). New therapeutic drugs are therefore needed.

MIRV was primarily tested in the single-arm SORAYA trial in FRα-positive platinum-resistant ovarian cancer. Median overall survival was 15 months, and objective response rate (ORR) was 32.4%. With 5 complete responses and 29 partial responses, MIRV showed promise in platinum-resistant ovarian cancer treatment (11).

In heavily pretreated patients with recurrent FRα-positive platinum-sensitive ovarian cancer, the single-arm phase II-PICCOLO trial showed benefit in therapy with MIRV after more than three systemic treatments before. ORR was shown to be 52%, and median progression-free survival (PFS) was 6.93 months (16).

The following MIRASOL study, phase 3, randomized controlled trial compared MIRV with chemotherapy of the physician’s choice in platinum-resistant ovarian cancer. Further lines of chemotherapy were allowed (1–3). Required was FRα expression on ovarian tumor tissue ≥ 75%. The primary endpoint was PFS with 5.62 months versus 3.98 months with chemotherapy. The ORR was significantly higher with 42.3% in the MIRV group than with chemotherapy (15.9%). Furthermore, the median overall survival was approximately 16.5 months (MIRV) versus 12.7 months (chemotherapy). Similar to the SORAYA trial, complete response was observed in the MIRV group with 12 patients. Partial response was seen in 84 patients. Progressive disease occurred more often in the chemotherapy group (27.4%) in comparison to MIRV (13.7%). Because of strong results with MIRV in platinum-resistant advanced ovarian cancer, MIRV was approved by the U.S. Food and Drug Administration (FDA) in the United States (1).

Subsequently, we tried to get MIRV for our patients with advanced relapsed ovarian cancer and high expression of FRα (≥75%) as off-label therapy.

Between July 2023 and August 2024, we could treat six patients with MIRV off-label in our Comprehensive Cancer Center.

## 2 Methods

This retrospective single-center cohort study was conducted after obtaining informed patient consent between July 2023 and August 2024. We could include six patients with metastatic advanced high-grade serous ovarian cancer (HGSOC) or high-grade serous primary peritoneal carcinoma (PPC) with expression of FRα ≥75% in malignant ovarian tissue or metastasis and progressive disease in computed tomography (CT) scan at the University Medical Center Freiburg (Comprehensive Cancer Center). Expression of FRα was detected with immunohistochemistry (IHC) and with the PS2+ scoring system. Membranous staining was categorized with an intensity scale (0 to 3+). High expression was defined as ≥75% (PS2+) of the tumor cells showing membranous staining.

Inclusion criteria were metastatic advanced HGSOC or PPC, expression of FRα ≥75%, and progression during systemic therapy with ≥3 therapies before. Patient selection is illustrated in **Table 1**.

**Table 1:** Patient characteristics.

Characteristic	Patient A	Patient B	Patient C	Patient D	Patient E	Patient F
Age at initial diagnosis (years)	39	55	58	70	61	58
Age at first therapy with Mirvetuximab (years)	49	63	60	73	63	59
Primary cancer diagnosis	HGSOC (High-grade serous ovarian cancer)	HGSOC	HGSOC	High-grade serous primary peritoneal carcinoma (PPC)	HGSOC	HGSOC
Stage at initial diagnosis	FIGO IIIC	FIGO IIC	FIGO IIIC	FIGO IV	FIGO IIIC	FIGO IVB
Metastasis	Cutan, lymphonodal, cerebral	Relapse with hepatic, peritoneal, lymphonodal metastasis	Peritoneal	Peritoneal, pleural, hepatic, lymphonodal	Peritoneal, subcutan, hepatic	Lymphonodal, hepatic, gastric
Race	White	Asian	White	White	White	White
ECOG at baseline	1	1	0	2	1	0
BMI at baseline (kg/m^2^)	23.9	22.4	20.9	24.4	24.5	25.1
Comorbidities	–	Hypothyreosis	Hypertension	Severe valvular aortic stenosis Paroxysmal atrial fibrillation	–	–

Demographic, diagnostic, clinicopathological, and treatment data were obtained from the hospital’s digital documentation system (Prometheus) and the tumor registry. Imaging was performed with a CT scan of the thorax–abdomen. During systemic therapy, CT scan was conducted every 3 to 6 months. All suspicious findings were recorded.

For statistical analyses, Microsoft Excel 2010 (Microsoft, Redmond, WA, USA) was used. Data are presented as means. Qualitative parameters are presented as absolute numbers and percentages. Clinical trial number: not applicable. Human ethics was approved by the Ethics Committee of the University Freiburg (25-1375-S1-retro).

## 3 Results

### 3.1 Patient characteristics

Our patients were diagnosed with advanced HGSOC or high-grade serous PPC with initial FIGO stage IIC–IV. The initial median age at diagnosis was 56.8 years and age at first therapy with MIRV was 61.2 years, as seen in **Table 1**. They showed good general condition with a median ECOG performance status score of 1 and a body mass index (BMI) at baseline of 20.5 kg/m^2^. Comorbidities like arterial hypertension and hypothyroidism were seen. One patient showed severe valvular aortic stenosis; therefore, she underwent transcatheter aortic-valve implantation (TAVI), and paroxysmal atrial fibrillation was maintained (**Table 1**).

All patients underwent surgery, primary debulking surgery, or interval debulking surgery with resection status R0 to R2. Chemotherapy was received as neoadjuvant, adjuvant, or during progression of disease. Median number of previous lines of therapy was 5.5, including chemotherapy, maintenance therapy with PARP inhibitors, or endocrine therapy, as shown in **Table 2**.

**Table 2:** Previous treatment.

Characteristic	Patient A	Patient B	Patient C	Patient D †	Patient E	Patient F
No. of previous lines of therapy	6	8	4	8	3	4
Chemotherapy	6× adj. C/T 6× Carboplatin/Caelyx 4× Carboplatin/Gemcitabine 6× Carboplatin/Gemcitabine (reinduction) 1× Topotecan	6× adj. C/T Carboplatin/Gemcitabine (unknown cycles) Carboplatin mono (unknown cycles) 4× Carboplatin mono (reinduction) 3× Caelyx 4× Topotecan 5× Trastuzumab-Deruxtecan	4× NACT C/T, 2× adj. C/T 3× Carboplatin/Caelyx 6× Gemcitabine	3× NACT C/T 6× Caelyx 6× Gemcitabine 9× Topotecan 3× Treosulfan 3× Vinorelbin	3× NACT C/T with PD 6× Caelyx 3× Topotecan	3× NACT C/T 3× adj. C/T 3× Caelyx 1× Topotecan
PARB inhibitor	Niraparib 36 months	Olaparib 23 months	Niraparib 6 months	–	–	Niraparib 4 months
Bevacizumab	X (in combination with C/T)	–	X (in combination with Gemcitabine)	X (in combination with C/T)	–	X (in combination with Caelyx)
Pembrolizumab	–	–	–	X	–	–
Endocrine therapy	–	–	–	X	–	–
Primary platinum-free interval (months)	27	35	6	–	PD at NACT	4
BRCA/HRD-Status	Neg/Pos.	Neg/unknown	Neg/neg.	Neg/neg.	Neg/neg.	Neg/neg.
Surgery (resection status)	Primary debulking surgery (R2)	Primary debulking surgery (R0), Relapse-surgery (R1)	Interval debulking surgery (R0)	Interval debulking surgery (inoperable)	Interval debulking surgery (R2)	Interval debulking surgery (R0)

adj., adjuvant, NACT, neoadjuvant chemotherapy, C/T, Carboplatin/Taxol (Paclitaxel), Caelyx, pegylated liposomal doxorubicin, PD, progressive disease, R0/1/2, resection status, R0, surgical margin microscopically negative, R1, surgical margin microscopically positive and macroscopically negative, R2, surgical margin micro- and macroscopically positive. The cross marks died people.

After primary surgery, adjuvant chemotherapy with six cycles of carboplatin/paclitaxel (*n*, 3) was given. Interval debulking surgery was done after three neoadjuvant cycles of carboplatin/paclitaxel (*n*, 3). Primary platinum-free interval was very different from 4 to 35 months. Indeed, two patients showed progress during neoadjuvant chemotherapy (**Table 2**).

Primary or in progression metastasis occurred, mostly as peritoneal, lymphonodal, hepatic, and sub-/cutan. Gastric and cerebral metastasis were rare.

Chemotherapy in progressive disease was conducted with or without carboplatin, depending on the primary platinum-free interval and platinum sensitivity. Three patients proved to be platinum-resistant (patients D, E, and F); they received mono-chemotherapy with pegylated liposomal doxorubicin (caelyx), topotecan, treosulfan, or vinorelbin. The group of platinum-sensitive patients received maintenance therapy with Niraparib or Olaparib, depending on BRCA and HRD status. In progress, we recommended poly-chemotherapy with carboplatin/caelyx, carboplatin/gemcitabine, or topotecan. Off-label, with overexpressed HER2/neu status, one patient could receive Trastuzumab-Deruxtecan off-label (**Table 2**).

### 3.2 Clinical experience and management

After all therapies according to our guidelines, we saw progression of the disease in CT scan of the thorax–abdomen; therefore, we looked to off-label therapies like MIRV. We measured the expression of FRα in malignant tissue (primary tumor or metastasis). With the expression of FRα ≥75% (IHC PS2+) and progressive disease, the health insurance should cover the treatment cost after the patient provides consent.

MIRV was first used in advanced platinum-resistant ovarian cancers (MIRASOL and SORAYA). Three patients proved to be platinum-resistant; the other half of the patients were platinum-sensitive (patients A, B, and C), but progressed after various lines of therapy. Therefore, we tried to treat them with MIRV as well, although MIRV was first administered for platinum-resistant ovarian cancers.

Before starting therapy with MIRV, our patients underwent ophthalmological examination, including visual acuity and slit lamp examination. To prevent ocular toxicity, our patients received prophylactic lubricating and glucocorticoid eye drops before and several days after treatment.

MIRV (Elahere ^®^) was received intravenously at a dose of 6 mg/kg adjusted to ideal body weight (AIBW) every 3 weeks (q3w) (12).

Before every therapy, we calculated the individual total dose of MIRV AIBW (12):


AIBW=Ideal Body Weight (IBW [kg]+0.4×(Actual weight [kg]−IBW) 

Female IBW [kg]=0.9×height [cm]−92

Two patients received dose reduction due to comorbidities, and the median dose was approximately 89% (see **Table 3**).

**Table 3:** Application of MIRV and treatment response.

Characteristic	Patient A	Patient B	Patient C	Patient D †	Patient E	Patient F
Prior platinum response	Platinum-sensitive			Platinum-resistant		
Received cycles of MIRV	3	3	2	2	4	6
Doses (6 mg/kg adjusted ideal body weight (AIBW), total amount)	75% (= 372, 82 mg)	100% (= 305, 76 mg)	100% (= 355, 32 mg)	60% (= 219, 31 mg)	100% (= 317, 7 mg)	100% (= 432, 6 mg)
CA-125 Baseline	547 U/mL	*	533 U/mL	402 U/mL	68 U/mL	1607 U/mL
CA-125 after 2–6 cycles of MIRV	471 U/mL	*	174 U/mL	†	31 U/mL	241 U/mL (after 3 cycles) 421 U/mL (after 6 cycles)
Response (CT thorax–abdomen)	SD	SD	PR	†	PD	PR
Toxicity		Hyperbilirubin-emia (PTCD) (CTCAE grade 3)	Microcystic keratitis, cataracta corticonuclearis incipiens (CTCAE grade 3)	Sudden cardiac death at home		Polyneuropathy (CTCAE grade 1), blurred vision

(Descriptive radiographic assessment, non-RECIST-based).

PR, Partial response, SD, Stable disease, PD, Progressive disease.

*Patient B: non-available data for CA-125 baseline and during therapy with MIRV.

CTCAE (Common Terminology Criteria for Adverse Events) grades:

Grade 1: Asymptomatic or mild symptoms; clinical or diagnostic observations only; intervention not indicated.

Grade 2: Moderate; minimal, local, or noninvasive intervention indicated; limiting age-appropriate instrumental (ADL).

Grade 3: Severe or medically significant but not immediately life-threatening; hospitalization or prolongation of hospitalization indicated; disabling; limiting self-care ADL.

Grade 4: Life-threatening consequences; urgent intervention indicated.

Grade 5: Death related to AE. The cross marks died people.

Premedication with paracetamol, dexamethasone, clemastine, and ondansetron was applied.

**Figure 1:**
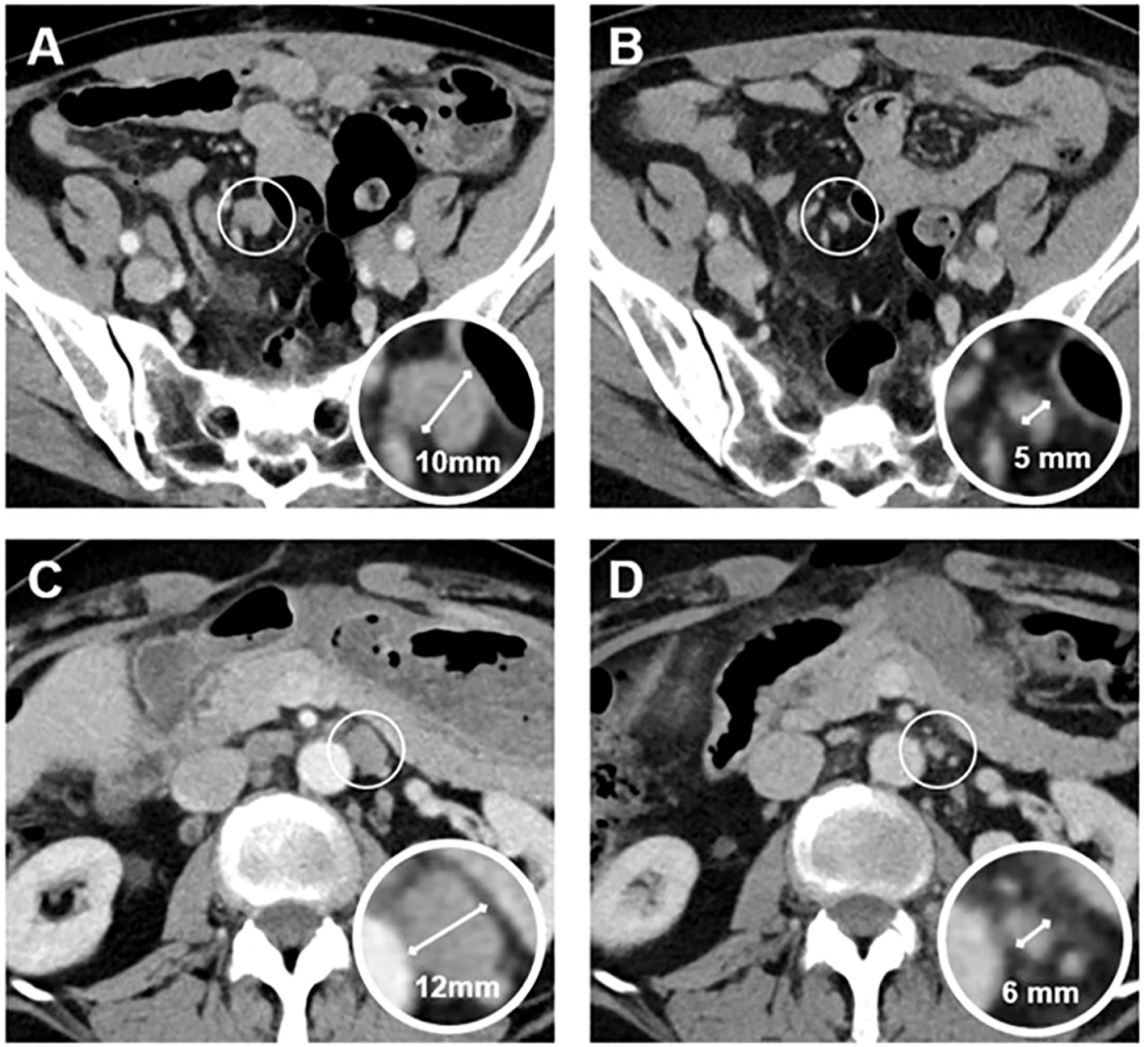
Contrast-enhanced axial abdominal CT images of a 59-year-old woman with platinum-resistant ovarian cancer, acquired before **(A, C)** and after **(B, D)** six cycles of treatment with Mirvetuximab-Soravtansin. The images and corresponding magnified views demonstrate mesenteric **(A, B)** and paraaortic **(C, D)** lymph node metastases. Post-treatment images **(B, D)** show normalization of lymph node size, as indicated by measurements.

After 2–6 cycles of MIRV (median, 3.3), objective descriptive response with CT scan of the thorax–abdomen (Non-RECIST-based) and CA-125 response were monitored. In every patient, we could measure CA-125 (data not available for patient B), we saw a reduction of CA-125 from baseline, as shown in **Table 3**. In CT scan of the thorax–abdomen, two patients showed stable disease with platinum-sensitive ovarian cancer. One of our patients with platinum-resistant ovarian cancer represented progressive peritoneal and hepatic disease (patient E). Two platinum-resistant patients revealed partial radiographic responses, one of whom had partial remission at initial FIGO stage IV (patient F) (see **Table 3** and **Figure 1**). We saw radiological response with lymph nodes and hepatic metastasis getting smaller. Gastric metastases were no longer seen. Owing to partial remission in the first CT scan after three and six cycles and clinical benefit, we continued therapy with MIRV until today. Partial remission was seen in regressive mesenteric and paraaortic lymph nodes (see **Figure 1**). MIRV was tolerated well; polyneuropathy was preexisting from previous therapies (CTCAE grade 1, asymptomatic). Irregular blurred vision occurred, and lubricating and glucocorticoid eye drops were used to improve vision. Ophthalmological examinations were conducted regularly.

One patient (patient D) with cardiac comorbidities and ECOG, 2 (at baseline) suffered sudden cardiac arrest at home after two cycles of MIRV (12 days after last infusion), most likely due to a pre-existing condition (**Tables 1**, **3**).

Patient B developed hyperbilirubinemia grade 3 (CTCAE criteria), defined as elevation of blood bilirubin >3.0–10.0 × ULN (upper limit of normal) with a normal baseline. Progressive hepatic metastasis led to biliary strictures with the need for percutaneous transhepatic biliary drainage. Additional adverse reaction of MIRV could not be excluded (**Table 3**). Therapy with MIRV could not be continued due to hyperbilirubinemia and deterioration of the general condition.

One patient (patient C) developed ocular toxicity during therapy with MIRV. After two cycles, visual impairment and blurred vision were reported by our patient (**Table 3**). Our colleagues from the Department of Ophthalmology described microcystic keratitis with cataracta corticonuclearis incipiens and change of refraction (CTCAE grade 3). CTCAE grade 3 shows symptoms and a decrease in visual acuity, leading to limiting self-care. Ophthalmological therapy with topical steroids and eye drops, and close visual monitoring were conducted in the Department of Ophthalmology. During the ophthalmological treatment the oncological treatment was interrupted. After the two received cycles of MIRV, we could observe a partial response in CT scan and a simultaneous decline of tumor marker CA-125 (**Table 3**). After 2 months of treatment, visual impairment resolved and visual acuity normalized. Therefore, we recommended continuing with MIRV in dose reduction, which was declined by our patient.

## 4 Discussion

MIRV was first approved by the FDA in November 2022 for the treatment of FRα-expressing platinum-resistant ovarian cancer. In March 2024, the FDA granted full approval for MIRV for the treatment of adult patients with FRα-expressing platinum-resistant epithelial ovarian, fallopian tube, or primary peritoneal cancer treated with up to three prior therapies (13, 14). In November 2024, the European Medicines Agency (EMA) approved MIRV (Elahere^®^) (15).

In our Comprehensive Cancer Center, we treated a heterogeneous group of patients with advanced FRα-expressing HGSOC or PPC with MIRV between July 2023 and August 2024. Half of the patients showed platinum-sensitive ovarian cancer with a primary platinum-free interval between 6 and 35 months. The other half suffered from progression during neoadjuvant chemotherapy or platinum-resistant HGSOC. Overall, 5.5 previous lines of therapy were received before MIRV was applied. Therefore, our cohort was significantly more pretreated and heterogeneous in comparison to the MIRASOL or SORAYA study cohort with one to three previous lines of systemic therapy.

Half of our patients were platinum-sensitive with SD and PR, underlining the results from the PICCOLO trial regarding the small size of patients in our cohort.

Adverse events like blurred vision (40.8%), keratopathy (32.1), or dry eye (28.0) are common during therapy with MIRV (56.0%) (1). In ophthalmological examination, corneal alterations like microcystic-like epithelial changes (MEC) could be observed (17, 18). FRα expression was found in the retina, in contrast to the corneal tissue, where MEC was detected (19). Therefore, ocular adverse events may result from DM4-payload molecule instead of FRα. Unspecific reaction with or without target effect on the eyes could be seen during therapy with other ADCs like Tisotumab Vedotin for advanced cervical cancer (18). Discontinuation of MIRV, as we needed to perform, was 1.8% (4 participants) in MIRASOL (1).

Before starting therapy with MIRV, an ophthalmological examination is highly recommended. Risk factors for dry eyes should be discussed with the patient, e.g., medications like antihypertensives (18, 20). Lubricating eye drops, e.g., Vismed^®^ EDO, and glucocorticoid eye drops, e.g., Monodex^®^ 1 mg/mL (dexamethasone), should be used daily. Every second cycle, ophthalmological examination (slit lamp and visual acuity) was performed (12, 15, 18).

Abdominal pain, constipation, diarrhea, or nausea could develop during treatment with MIRV (1). Our patients did not get impaired heavily by gastrointestinal symptoms; medication or supportive therapy was not necessary.

Hepatic impairment in advanced metastatic ovarian cancer, especially with hepatic metastasis, should be monitored before and during therapy. Adjustment of dose from MIRV is not recommended with mild hepatic impairment [total bilirubin > 1–1.5 × ULN and any AST (aspartate-aminotransferase) or total bilirubin ≤ ULN and AST > ULN]. One of our patients developed hyperbilirubinemia, CTCAE grade 3, and we stopped the therapy with MIRV, as recommended by the scientific information (21). Hepatobiliary disorders could develop during therapy. Hyperbilirubinemia and, therefore, therapeutic use of PTCD as we did are not yet described in the literature in association with MIRV to our knowledge.

Peripheral neuropathy appeared in 21.6% in MIRASOL (1). Most of our patients showed peripheral neuropathy already during therapy with taxane. One patient described symptoms of polyneuropathy, CTCAE grade 1, and we recommended supportive therapy. Peripheral neuropathy did not worsen during continuation of MIRV.

Cardiac toxicity of MIRV was not described in the literature or in the summary of product characteristics (12, 15). Therefore, we suggest that our patient with strong cardiac comorbidities suffered cardiac arrest at home independently of therapy with MIRV. In our retrospective cohort study, causality cannot be determined.

Further adverse events that we did not observe in our cohort should be known before and during treatment with MIRV. Hematological side effects, e.g., anemia, thrombocytopenia, and leukopenia, or lung-affecting adverse events like pneumonitis are reported (1, 11).

Complete response in advanced pretreated HGSOC is rare with chemotherapy, e.g., 0 patient in the chemotherapy group in MIRASOL in comparison to 12 in the MIRV group. In literature, one case report showed a complete response in FDG-PET in a heavily pretreated Asian patient (22). One of our patients showed partial remission with a significant reduction of the size of metastasis in lymph nodes and liver, as well as a reduction of the tumor marker CA-125. Because of good overall status and response, we continued therapy with MIRV until today. We have to admit that the measurement of CA-125 could not be performed in all patients.

In the future, MIRV could be a therapeutic option in the treatment of different subtypes of ovarian cancer. We treated one patient with high-grade serous PPC with high expression of FRα. In low-grade serous ovarian carcinoma (LGSOC), expression of FLOR1 (FRα) is detected in 30% (23). In the future, MIRV could be a therapeutic target in different subtypes of ovarian cancer beyond HGSOC, but further studies are needed.

MIRV proves to be a promising therapy in advanced metastatic ovarian cancer based on the results of SORAYA and MIRASOL. They demonstrated significantly better results than chemotherapy of the physician’s choice.

We have to admit that the general conclusion and recommendations based on the small sample size and the heterogeneous group of our patients are limited. We intended to show how to implement new therapies in clinical treatment. We could observe the reported results with partial response on stage IV ovarian cancer.

We worked interdisciplinarily with our colleagues from ophthalmology to prevent and treat ocular toxicity. Patient education should be done before treatment, as well as close interdisciplinary work to prevent adverse events.

After the approval of MIRV in Europe, more patients with advanced ovarian cancer can receive MIRV. Our experience and results from real-world data will grow. Various treatments could be applied in different subtypes of ovarian cancer. Further investigations of the expression of FRα in different tumor tissues are needed to expand the treatment possibilities of MIRV.

## Data Availability

The original contributions presented in the study are included in the article/supplementary material. Further inquiries can be directed to the corresponding author.
